# Association between functional gastrointestinal disorders and Parkinson’s disease in a prospective cohort study

**DOI:** 10.1038/s41531-025-01000-4

**Published:** 2025-06-04

**Authors:** Yixiang Lin, Haoling Xu, Jiayi Zheng, Tianxin Lin, Minhui Wang, Tingting Huang, Fabin Lin, Qinyong Ye, Guoen Cai

**Affiliations:** 1 Department of Neurology, Center for Cognitive Neurology, Institute of Clinical Neurology, Fujian Medical University Union Hospital, 29 Xinquan Road, Fuzhou, 350001 China; 2 Fujian Institute of Geriatrics, Fujian Medical University Union Hospital, 29 Xinquan Road, Fuzhou, 350001 China; 3 Fujian Key Laboratory of Molecular Neurology, Institute of Clinical Neurology, Institute of Neuroscience, Fujian Medical University, 88 Jiaotong Road, Fuzhou, 350001 China; 4 School of Basic Medical Sciences, Fujian Medical University, Fuzhou, 88 Jiaotong Road, Fuzhou, 350001 China; 5 Department of Neurosurgery, Fujian Medical University Union Hospital, 29 Xinquan Road, Fuzhou, 350001 China

**Keywords:** Diseases, Risk factors

## Abstract

The influence of functional gastrointestinal disorders (FGIDs) on the onset of Parkinson’s disease (PD) remains unclear. Therefore, in this study, we examined the effect of FGIDs and their subtypes on the PD onset. In Cox proportional hazards model, FGIDs significantly increased the risk of PD incidence [hazard ratio (HR) = 1.74, 95% confidence interval (CI) = 1.30–2.33]. Similar results were also observed for functional dyspepsia (HR = 1.71, 95% CI = 1.17–2.52) and other functional intestinal disorders (other FIDs) (HR = 1.67, 95% CI = 1.00–2.78). Mediation analyses revealed that mental health scores mediated 10.00% and 8.32% of the association between FGIDs and functional dyspepsia and PD development. This cohort study discovered that FGIDs increase the risk of developing PD. Similar effects can also be observed in functional dyspepsia and other FIDs and mental health mediates part of the effect of FGIDs and functional dyspepsia on PD.

## Introduction

Parkinson’s disease (PD) is a progressive chronic neurodegenerative disease associated with aging^1^. The main symptoms of PD are motor dysfunctions, such as tremors and bradykinesia. In addition, some patients exhibit nonmotor dysfunctions such as constipation^2–4^. The prevalence of PD is approximately 1–2 per 1000 in the general population and reaches 1% in individuals over 60 years of age^5^. In 2017, there were close to 1 million people with PD in the United States alone, which accounted for a total economic burden of $51.9 billion^6^. PD imposes a substantial socioeconomic burden, for which there is currently no effective treatment^7^. Therefore, identifying potential risk factors for PD and populations susceptible to PD at the earliest possible can help take measures to prevent its onset and avoid a significant public health issue.

Functional gastrointestinal disorders (FGIDs) are characterized by persistent gastrointestinal symptoms without any discernible pathology or abnormalities in the anatomy or physiology during routine assessment. However, there is a correlation with the patient’s mental state^8^. FGIDs are often accompanied by anxiety, depression, and other psychiatric disorders^9,10^. Moreover, anxiety and depression increase the risk of PD onset^11^; FGIDs might therefore exacerbate the risk of PD. The Braak hypothesis postulates that pathological alterations of α-synuclein propagate retrogradely from the gastrointestinal tract via the vagus nerve to the brainstem, ultimately reaching the substantia nigra pars compacta in the ventral midbrain. Within this region, these pathological aggregates preferentially target and degenerate dopaminergic neurons responsible for motor regulation, suggesting a potential gastrointestinal origin for PD pathogenesis^12,13^. Moreover, a past study demonstrated that mice receiving fecal microbiota transplantation from PD patients exhibited a significant reduction in dopaminergic neurons compared to control groups. The investigators propose that dysbiosis of the gut microbiome in PD disrupts both the healthy microbiota community and Th17-mediated homeostatic immunity in ileal mucosa. This pathological alteration may trigger a cascade of events that propagate to the brain, ultimately driving the pathogenesis of PD^14^.

Nonetheless, the effect of FGIDs on PD remains unclear. The link between irritable bowel syndrome (IBS), a type of FGID, and PD is not yet completely understood^15,16^. For example, a cohort study using data from the Finnish Care Register for Health Care noted that IBS aggravates the risk of developing PD^16^. However, another meta-analysis concluded that there was no significant association between them^15^. A similar debate is ongoing regarding the association between functional dyspepsia and PD. A matched study observed a lack of significant association between the two^17^. Another case–control study reported that functional dyspepsia may be a risk factor for PD^18^. Nonetheless, there are certain limitations in the aforementioned studies. For example, matching studies only consider a few variables, such as age, sex, ethnicity, and race, which can lead to biases because of the potential covariate. On the contrary, a limitation of the case–control studies is that they involve only a few participants. Therefore, considering the limitations and inconsistent results of the abovementioned studies, further investigation of the association between FGIDs and PD is warranted.

Thus, this research was designed with data sourced from a large-scale cohort from the UK Biobank to evaluate in-depth the effect of FGIDs on PD. The association between FGIDs and their subtypes and PD was examined by using the Cox proportional hazards model. A subgroup analysis using the polygenic risk score (PRS) for PD was performed to determine whether the effect of FGIDs on PD differs among different PD susceptibility groups. The role of mental health scores in the relationship between FGIDs and PD onset was evaluated through mediation analyses.

## Results

### Baseline characteristics of study participants

This study included a sample of 172,239 participants. Of these, 4615 participants had FGIDs before the baseline. The median follow-up period was 13.6 years, and 954 participants developed PD during the follow-up period. The participants with FGIDs at the baseline were elderly and more likely to be female. Moreover, they exhibited higher TDI, lower education levels, more numbers of diabetes patients, lower physical activity, and worse mental status relative to the healthy population (Table 1).

**Table 1:** Sample characteristics of participants according to functional gastrointestinal diseases

Variables	Overall	FGIDs		*P*-value^a^
	(N = 172239)	No	Yes	
		(*N* = 167624)	(*N* = 4615)	
Sex = Male (%)	81013 (47.0)	79405 (47.4)	1608 (34.8)	<0.001
Age (mean (SD))	54.95 (8.00)	54.91 (8.00)	56.40 (7.82)	<0.001
TDI (mean (SD))	−1.48 (2.98)	−1.49 (2.98)	−1.15 (3.17)	<0.001
Drinking status (%)				<0.001
Never	5945 (3.5)	5682 (3.4)	263 (5.7)	
Previous	5561 (3.2)	5286 (3.2)	275 (6.0)	
Current	160733 (93.3)	156656 (93.5)	4077 (88.3)	
Smoking state (%)				0.278
Never	69632 (40.4)	67814 (40.5)	1818 (39.4)	
Previous	85147 (49.4)	82839 (49.4)	2308 (50.0)	
Current	17460 (10.1)	16971 (10.1)	489 (10.6)	
Race (%)				0.032
Asia	2960 (1.7)	2891 (1.7)	69 (1.5)	
Black	867 (0.5)	844 (0.5)	23 (0.5)	
Mixed	684 (0.4)	668 (0.4)	16 (0.3)	
Others	3464 (2.0)	3343 (2.0)	121 (2.6)	
White	164264 (95.4)	159878 (95.4)	4386 (95.0)	
Hypertension (%)				<0.001
Normal	46939 (27.3)	45465 (27.1)	1474 (31.9)	
Elevated	36642 (21.3)	35661 (21.3)	981 (21.3)	
Stage 1 hypertension	31654 (18.4)	30813 (18.4)	841 (18.2)	
Stage 2 hypertension	57004 (33.1)	55685 (33.2)	1319 (28.6)	
Education (%)				<0.001
Junior high school	45368 (26.3)	44091 (26.3)	1277 (27.7)	
Higher school	21542 (12.5)	21037 (12.6)	505 (10.9)	
College	77056 (44.7)	75369 (45.0)	1687 (36.6)	
Other	28273 (16.4)	27127 (16.2)	1146 (24.8)	
BMI (%)				<0.001
Underweight	1020 (0.6)	968 (0.6)	52 (1.1)	
Health	61560 (35.7)	59919 (35.7)	1641 (35.6)	
Overweight	72198 (41.9)	70390 (42.0)	1808 (39.2)	
Obesity	37461 (21.7)	36347 (21.7)	1114 (24.1)	
Diabetes = Yes (%)	6427 (3.7)	6188 (3.7)	239 (5.2)	<0.001
At or above moderate vigorous walking recommendation = Yes (%)	140945 (81.8)	137323 (81.9)	3622 (78.5)	<0.001
Healthy diet score (%)				<0.001
0	3487 (2.0)	3405 (2.0)	82 (1.8)	
1	16842 (9.8)	16455 (9.8)	387 (8.4)	
2	36343 (21.1)	35414 (21.1)	929 (20.1)	
3	47012 (27.3)	45745 (27.3)	1267 (27.5)	
4	44243 (25.7)	43014 (25.7)	1229 (26.6)	
5	24312 (14.1)	23591 (14.1)	721 (15.6)	
Mental health score = High (%)	75671 (43.9)	73047 (43.6)	2624 (56.9)	<0.001
Polygenic risk scores (mean (SD))	−0.14 (1.02)	−0.14 (1.02)	−0.14 (1.01)	0.802

*FGIDs* Functional gastrointestinal disorders, *TDI* Townsend deprivation index, *BMI* Body mass index, *SD* Standard deviation.

^a^Analysis of variance or χ^2^ test where appropriate.

### Association between FGIDs and PD risk

In the Cox proportional hazards model, the results of Model 3 indicated that patients with FGID exhibited a higher susceptibility to developing PD than the healthy population (HR = 1.74, 95% CI = 1.30–2.33, *P*-value < 0.001). In the FGID subtypes, patients with functional dyspepsia were more likely to suffer from PD than the healthy population (HR = 1.71, 95% CI = 1.17–2.52, *P*-value = 0.006). Although in Model 1, patients with IBS were more likely to develop PD than the healthy population (HR = 2.03, 95% CI = 1.05–3.92, *P*-value = 0.034), Model 3 was only slightly short of significance (HR = 1.79, 95% CI = 0.93–3.46, *P*-value = 0.083). Patients with other FIDs had a 67% elevated risk of PD onset than the healthy population (HR = 1.67, 95% CI = 1.00–2.78, *P*-value = 0.050) (Table 2).

**Table 2:** Associations between functional gastrointestinal diseases and Parkinson’s disease

Items	FGIDs		Subtypes					
			Functional dyspepsia		IBS		Other FIDs	
	No	Yes	No	Yes	No	Yes	No	Yes
Participants	167624	4615	169893	2346	171286	953	170730	1509
Number of cases	905 (0.54%)	49 (1.06%)	927 (0.55%)	27 (1.15%)	945 (0.55%)	9 (0.94%)	939 (0.55%)	15 (0.99%)
Model1^a^	Ref	1.89 (1.42–2.53, *p* < 0.001)	Ref	1.84 (1.26–2.70, *p* = 0.002)	Ref	2.03 (1.05–3.92, *p* = 0.034)	Ref	1.82 (1.09–3.03, *p* = 0.022)
Model2^b^	Ref	1.81 (1.36–2.42, *p* < 0.001)	Ref	1.78 (1.22–2.62, *p* = 0.003)	Ref	1.90 (0.98–3.66, *p* = 0.058)	Ref	1.72 (1.03–2.87, *p* = 0.037)
Model3^c^	Ref	1.74 (1.30–2.33, *p* < 0.001)	Ref	1.71 (1.17–2.52, *p* = 0.006)	Ref	1.79 (0.93–3.46, p = 0.083)	Ref	1.67 (1.00–2.78, *p* = 0.050)

Results were presented HR and 95% CI.

^a^Model 1 was adjusted for age, sex, TDI, race and education level.

^b^Model 2 was further adjusted for smoking statue, drinking status, blood pressure, diabetes, BMI, physical activity, healthy diet scores.

^c^Model 3 Further adjusted the mental health scores based on model 2.

### Association between FGIDs and PD after stratifying the genetic risk of PD

**Fig. 1:**
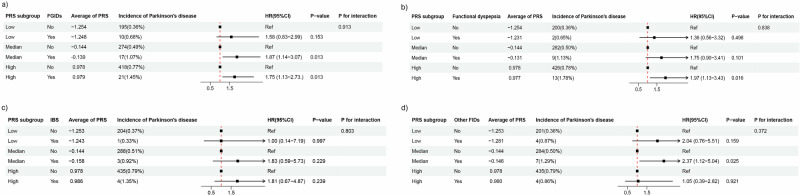
Association between functional gastrointestinal disorders and their subtypes and Parkinson's disease in groups stratified by polygenic risk score. Association between **a** functional gastrointestinal disorders, **b** functional dyspepsia, **c** irritable bowel syndrome and **d** other functional intestinal disorders and Parkinson’s disease after grouping participants using polygenic risk score for Parkinson’s disease.

The PRS of PD was used to categorize participants into low, medium, and high susceptibility groups to developing PD. No significant interactions were observed between exposure variables and PRS in the FGIDs (P for interaction = 0.913), functional dyspepsia (P for interaction = 0.838), IBS (*P* for interaction = 0.803), and other FIDs (*P* for interaction = 0.372) (Fig. 1). These observations implied that the effect of FGIDs on PD onset was similar in populations with varying susceptibilities to PD.

### The mediating role of mental health score in the association between FGIDs and PD incidence

**Fig. 2:**
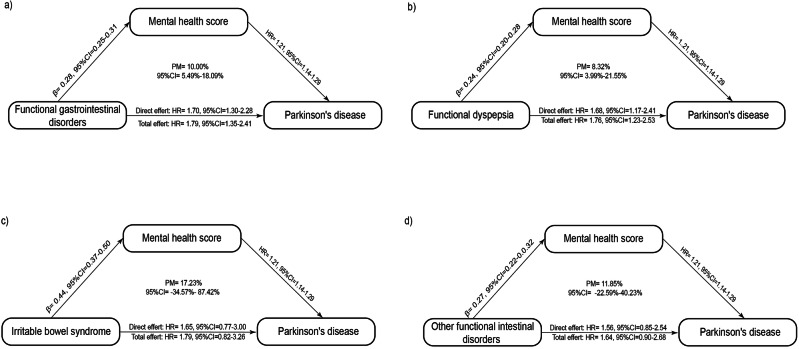
Mediating effects of mental health score in the association of functional gastrointestinal disorders and their subtypes with the onset of Parkinson's disease. Mediation analysis of the role of mental health score in the association between (**a**) functional gastrointestinal disorders, **b** functional dyspepsia, **c** irritable bowel syndrome and (**d**) other functional intestinal disorders and the onset of Parkinson’s disease.

For every unit increase in the standardized mental health score, a corresponding increase was noted in the risk of PD development (HR = 1.21, 95% CI = 1.14–1.29). Both patients with FGIDs (β = 0.28, 95% CI = 0.25–0.31) and those with functional dyspepsia (β = 0.24, 95% CI = 0.20–0.28) exhibited increased mental health scores when compared with the healthy population. Mediation analyses revealed that standardized mental health scores mediated 10.00% (95% CI = 5.49%–18.09%) and 8.32% (95% CI = 3.99%–21.55%) of the association in the effect of FGIDs and functional dyspepsia, respectively, on PD onset (Fig. 2).

### Sensitivity analysis

When plotting the cumulative incidence curves, the PD incidence curves of FGIDs (*p* < 0.001) differed significantly from those of the healthy population. A similar result was observed between functional dyspepsia (*P* < 0.001) and other FIDs (*P* = 0.013). However, the PD incidence curve in IBS (*P* = 0.098) was not significantly different from that of the healthy population (Supplementary Fig. S1). The results of subgroup analyses were broadly stable after analyzing the subgroups for age (*P* for interaction = 0.197), sex (*P* for interaction = 0.790), TDI (*P* for interaction = 0.858), alcohol consumption (P for interaction = 0.895), smoking (P for interaction = 0.810), BMI (*P* for interaction = 0.598), blood pressure status (*P* for interaction = 0.979), healthy diet score (*P* for interaction = 0.737), diabetes mellitus (*P* for interaction = 0.785), exercise status (*P* for interaction = 0.478), and mental health score (*P* for interaction = 0.269). This finding signified that the results from this investigation could be generalized to a broad range of populations, extending beyond the specific characteristics (Supplementary Table S1). Furthermore, the Cox proportional hazards model was repeated after eliminating participants with a PD diagnosis of <2 or 4 years, and the results agreed with those from the main analysis (Supplementary Table S2 and Supplementary Table S3). The number of participants reached 206,418 after adding those with missing exercise data using multiple imputations. The analysis was repeated, and the results remained the same (Supplementary Table S4). The link between FGIDs and the onset of PD was re-evaluated after excluding participants with FGIDs after the baseline. However, the observations were considerably similar to the previous results (Supplementary Table S5). When the number of boots was 500, the mediation analysis resembled the earlier results. Hence, the robustness of the findings from the mediation analysis was established (Supplementary Fig. S2). No significant interaction was observed when the PRS format in the interaction term was continuous or dichotomous variables. The results were comparable to previous results (Supplementary Table S6). The association between FGIDs and PD remained consistent with the primary findings in the time-dependent Cox model. While marginal adjustments in HRs were observed, both the statistical significance level and the direction of effect remained materially unchanged, demonstrating the robustness of our primary conclusions (Supplementary Table S7).

## Discussion

The outcomes of this prospective cohort study imply that FGIDs significantly increased the risk of PD development. Of the various FGID subtypes, functional dyspepsia and other FIDs were significantly related to an elevated risk of PD onset. However, the association between IBS and PD was only close to being significant. The effects of FGIDs and their subtypes on PD were similar in different PD-susceptible populations. In mediation analyses, mental health scores mediated the effects of FGIDs and functional dyspepsia on PD.

Only a few studies have reported the association between FGIDs and PD. Most studies have only examined the effect of a single type of FGID on PD. The effect of several specific FGIDs, including functional dyspepsia and IBS, on PD is not currently conclusive. One case–control study observed that dyspepsia might be a possible risk factor for PD^18^, which agrees with our findings. In contrast, another matched study concluded that there was no significant association between dyspepsia and PD^17^. One possible explanation for this discrepancy is that the matched studies considered only the variables of age, sex, race, and ethnicity when they matched healthy individuals with patients with dyspepsia. Variables not accounted for in matched studies might have contributed to the biased results. Moreover, there are mixed findings on the association between IBS and PD. A Finnish cohort study suggested that IBS increases the risk of developing PD^16^. The same conclusion was reached in a meta-analysis^19^. In contrast, our cohort study noted an association between IBS and PD slightly outside the significance level. This discrepancy could be attributed to the fact that the Finnish population-based cohort study considered very few covariates, with only six covariates, such as age and sex, and the meta-analysis included only six articles, with only 58,645 participants in the analysis. Nonetheless, another meta-analysis observed that IBS did not increase the possibility of acquiring PD^15^. The results support our conclusions. However, we differed slightly from another paper that also used UK Biobank to explore the association between IBS and PD^20^. The results of this study suggest that IBS reduces the risk of PD, which may be attributed to the different data sources and covariate selection for IBS. However, no significant results were observed in either study. In the future, larger cohort studies would be needed to validate the true association between the two factors. Only a few studies have investigated the effect of other FIDs on PD. However, the current research identified that constipation, a type of other FIDs, increases the risk of developing PD^21,22^.

PRS was applied to categorize the participants into three groups: low, moderate, and high PD susceptibility. PRS did not interact significantly with either FGIDs or their subtypes. This finding demonstrates that the effect of FGIDs on PD is the same across different PD-susceptible populations. The sensitivity study examined the interaction between covariates and PD using subgroup analyses. The results showed that the covariates did not interact with FGIDs. This observation indicates that the findings from this study can be applied to a wide range of populations, extending beyond specific features.

No large-scale study has so far explored the potential role of mental health status in the association between FGIDs and PD. Mediation analysis revealed that patients with FGIDs or functional dyspepsia had higher mental health scores than the healthy population. As the mental health score increases, the corresponding risk of developing PD also increases. The results suggest that poor mental status may mediate some of the effects of FGIDs or functional dyspepsia on PD. Another study showed that patients with PD have higher Hospital Anxiety and Depression Scale scores compared to the healthy population, indicating that people with PD are more likely to have a poor mental condition^23^. Another study based on a European population noted that depression may be linked to an increased risk of developing PD^24^. In addition, the current research suggests that worse mental status may be related to the onset of functional dyspepsia^25,26^.

This study has the following advantages: First, data from this study were collected from the large-scale prospective cohort UK Biobank. The model was adjusted for several covariates that may influence the outcome to avoid bias. In addition, the use of national registries for PD diagnosis ensured the accuracy and reliability of the results. However, this study has certain limitations: (1) The UK Biobank recruited mostly British participants and a few from other ethnic groups. Hence, further studies must confirm whether the findings from this research can be extrapolated to other races. (2) As functional diseases, FGIDs may be underestimated and under-recognized in the UK Biobank. This could result in potential classification errors and weaken the findings, thereby underestimating the genuine effect of FGIDs on PD. (3) This study categorized FGIDs into three main subtypes (functional dyspepsia, IBS, and other FIDs) and observed similar results as in FGID subtypes. Owing to the broad classification of FGIDs, it is necessary to interpret the results cautiously. (4) Some patients with FGIDs already had a long history of FGIDs before inclusion in the study, which may have led to a bias in the duration of the effect of FGIDs on PD. (5) Because mediation analyses derived from observational data are inherently hypothetical, the mediating pathways identified in this study should be interpreted as underlying mechanistic hypotheses rather than definitive causal chains. Subsequent using causal inference methods are needed to confirm this aspect. Subsequent studies are needed to confirm this finding using causal inference methods.

Our cohort study observed that FGIDs increase the risk of PD onset. Of the various subtypes of FGIDs, functional dyspepsia, and other FIDs were observed to be associated with a high risk of PD. Moreover, the relationships of FGIDs or their subtypes with PD were the same across PD-susceptible populations. In mediation analyses, the mental health score mediated the effects of FGIDs and functional dyspepsia on PD.

## Methods

### Study designs and participants

The UK Biobank is a large prospective cohort study that contains health-related data of more than 500,000 individuals across the UK. Participants aged 37–73 years were initially recruited during 2006–2010. The participants’ baseline data were completed via responses to survey forms, interviews, physical examinations, and genetic and biochemical tests^27^. The North West UK Multicentre Research Ethics Committee, the National Information Management Board, and the Government of the United Kingdom have approved the UK Biobank. A review was conducted for studies involving human participants. Written informed consent was obtained from the study patients/participants.

**Fig. 3:**
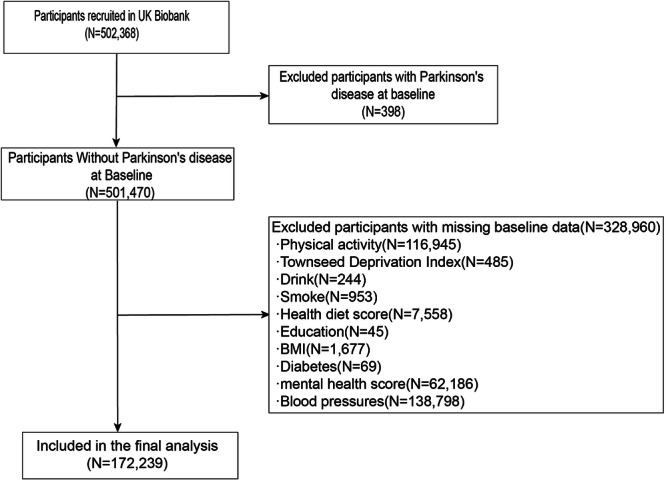
Selection of study participants in the UK Biobank.

Initially, this study enrolled 502,368 participants from the UK Biobank. However, 398 participants who had PD before the baseline were excluded. After the final exclusion of participants lacking baseline data, this study involved a total of 172,239 participants (Fig. 3).

### Definition of functional gastrointestinal disorders and PD

In this study, the participants were categorized into control and exposed groups based on whether they had FGIDs at the baseline. In addition, we further classified FGIDs into functional dyspepsia, IBS, and other functional intestinal disorders (other FIDs) as per a previous study^28^. Considering that FGIDs are more difficult to diagnose, the disease data in this study were selected from the hospitals with the highest level of confidence. Moreover, these records are based on the International Classification of Diseases, 10th edition (ICD-10), codes of K30 (dyspepsia), K58 (IBS), and K59 (other FID) codes. PD was defined in the same way as FGIDs.

The effect of FGIDs on the onset of PD was the primary outcome of this research. and the secondary outcome was the effect of the three FGID subtypes (including functional dyspepsia, IBS, and other FIDs) on PD.

### Definition of PRS for PD

The PRS is estimated to quantify the relative risk of developing a specific disease^29,30^. UK Biobank’s PRS was calculated using the Bayesian approach^31^. The PRS was trained only on external GWAS data and on ancestors from several different pedigree sources. Tests were also conducted in different cohorts to verify the robustness of the PRS, and the results were found to be similar across cohorts^32^. In addition, the GWAS data used by UK Biobank to calculate PRS was uploaded into the Zenodo database^33^. The higher the PRS, the more likely the participants were to develop PD. Depending on the trisection of their PRS, the participants were characterized to have low, medium, or high prevalence risk for PD.

### Mediator variable

The questions on the questionnaire were used to calculate the mental health score as per previous research, and based on mood fluctuations, distress, irritability, sensitivity to injured sentiments, tension, and other mental states (Supplementary Text S1). The higher the score, the worse was the mental state^28,34^. The mental state scores were standardized to be included as mediator variables in mediation analyses. This standardization effectively reduced the impact of extreme values on the results^35^. The standard score of the mental state scores was calculated as follows: standard score = original value of the mental state score − average of mental state score)/standard deviation of the mental state score.

### Assessment of covariates

The questionnaire via a touchscreen was employed to obtain participants’ baseline data, including age, sex, and race (White, Asian, Black, mixed, and others), smoking status (never, previous, and current), drinking status (never, previous, and current), educational level (college, higher school, junior high school, and others), and physical activity (whether participants fulfilled the UK guideline recommendation of at least 150 min of moderate-intensity exercise or 75 min of vigorous-intensity exercise per week). Townsend Deprivation Index (TDI), an indicator of socioeconomic status, was calculated from the residential zip codes by integrating data on car ownership, employment status, home ownership, and household density. The higher the index, the poorer the participant. Diabetes status was determined during the initial visit, with participants reporting a medical diagnosis of diabetes by a healthcare professional. Blood pressure readings were obtained using the Omron HEM-7015IT digital blood pressure monitor by averaging two seated measurements. According to the American Heart Association 2017 guidelines^36^, blood pressure was categorized into the following four groups: normal (systolic blood pressure (SBP) < 120 mmHg and diastolic blood pressure (DBP) < 80 mmHg), elevated (SBP 120–129 mmHg and DBP < 80 mmHg), stage 1 hypertension (SBP 130–139 mmHg or DBP 80–89 mmHg), and stage 2 hypertension (SBP ≥ 140 mmHg or DBP ≥ 90 mmHg). Furthermore, during the initial visit, trained professionals measured the participants’ height and weight to calculate the body mass index (BMI), which was classified as follows: underweight (BMI < 18.5), healthy (BMI 18.5 to <25), overweight (BMI 25 to <30), and obesity (BMI ≥ 30). The healthy diet score was derived from the weekly consumption of vegetables, fruit, fish, and meat on a scale of 0–5, with higher scores indicating a healthier diet^37^. According to the median mental health score, the participants were categorized into low and high groups. Specific measurement dates for the abovementioned covariates are provided in Supplementary Table S8.

### Statistical analysis

To determine the differences in the baseline characteristics between the normal participants and patients with FGIDs, the chi-squared (χ^2^) test was employed for categorical variables and the analysis of variance for continuous variables. The duration of follow-up was calculated from the date of the participant’s first visit to the study center to the earliest of several potential endpoints: the date of diagnosis of PD, the date of death, the date of loss to follow-up, or the date of the last available hospital admission for England (October 31, 2022), Scotland (August 31, 2022), and Wales (May 31, 2022).

Initially, a Cox proportional hazards model was employed to quantify the association between FGIDs or their subtypes and PD. The results were furnished using hazard ratios (HR) and 95% confidence intervals (CI). Three sets of models were used for the analysis. Age, sex, TDI, race, and education were adjusted in Model 1. Model 2 extended these adjustments to include variables such as smoking status, drinking status, blood pressure, diabetes, BMI, level of physical activity, and healthy diet score. In Model 3, the mental health score based on Model 2 was additionally included. The model’s assumption of proportional hazards was tested by constructing the interaction terms of time and FGIDs and the Schoenfeld residual method. According to the results, the model satisfied the proportional hazards assumption (Supplementary Table S9). Subsequently, the participants were trisected based on their PRS for PD, and a subgroup analysis was performed to determine the differential impact of FGIDs on PD across groups with varying susceptibilities to PD. The Cox proportional hazards model was applied based on the “survival” package in the R software^38^.

To examine whether the mental health score indirectly mediated the effect of FGIDs on PD, mediation analyses were performed based on the 2-stage regression method for survival data^39^. In mediation analyses, the coefficient “a” represented the association between the exposure factor and the mediator variable, “b” signified the link between the mediator variable and the outcome factor, and “c” denoted the total effect of the exposure on the outcome. Based on previous research, indirect effects were defined as a×b, with the proportion of mediation represented by a×b/c in this study^40^. A total of 200 boots were applied in mediation analyses based on previous studies^41^. These analyses were conducted after adjusting for potential confounding variables, including age, sex, TDI, race, education, smoking status, drinking status, blood pressure, diabetes, BMI, physical activity, and healthy diet score. We then drew directed acyclic graphs to visualize the causal associations between the variables based on previous studies (Supplementary Fig. S3). The mediation analyses were implemented through the “plyr” and “survival” packages in the R software^38,42^.

Moreover, the following sensitivity analyses were performed: (1) cumulative incidence curves were applied to examine whether the incidence of PD differed between healthy populations and patients with FGIDs. (2) The effect of FGIDs on PD was reassessed by using the Cox proportional hazards model after excluding patients with PD diagnosed less than 2 or 4 years ago. (3) Subgroup analyses were performed to evaluate possible interactions between covariates and FGIDs among participants. (4) A high number of participants were excluded due to missing exercise data; hence, multiple imputation was employed to complete the exercise data for those with missing information. The Cox proportional hazards model was used again to evaluate the effect of FGIDs on PD. Moreover, multiple imputation was performed with the “Mice” package in the R software^43^. (5) After excluding participants in the healthy population who developed FGIDs after the baseline, the Cox proportional hazards model was used to analyze the effect of FGIDs on PD. (6) To ensure the robustness of the results, the number of boots used again for the mediation analysis was 500. (7) We then transformed the PRS into dichotomous and continuous variables to construct interaction terms with the FGIDs separately so as to explore whether the interaction changed significantly. (8) We employed a time-dependent covariate approach to evaluate potential temporal variations in the association between FGIDs and PD. Specifically, the midpoint of the entire follow-up period (January 1, 2016) was designated as the secondary follow-up time point. Participants who developed FGIDs after baseline but before January 1, 2016, were dynamically reclassified into the disease group during the subsequent follow-up phases. Using this time-updated classification, we implemented a time-dependent Cox proportional hazards model to investigate whether the effect of FGIDs on PD incidence changed significantly.

All statistical analyses were performed using the R software version 4.4.1. A two-sided *p* < 0.05 was considered to indicate statistical significance. And Strengthening the Reporting of Observational Studies in Epidemiology (STROBE) was used as reporting guidelines (Supplementary Table S10).

## Supplementary information

Supplementary file

## Data Availability

Data obtained from the UK Biobank are available on application at www.ukbiobank.ac.uk/register-apply (94166).

## Code availability

All statistical analyses in this study were performed using R software version 4.4.1. The analytic methods, code, and study materials that support the findings of this study will be available from the corresponding authors on request.
