# Supplementary material for: Association between functional gastrointestinal disorders and Parkinson’s disease in a prospective cohort study
Source: NPJ Parkinsons Dis. 2025 Jun 4;11:150. doi: 10.1038/s41531-025-01000-4 (PMC12137633; doi:10.1038/s41531-025-01000-4)
Supplement: Supplementary file 1 — Supplementary file [file 41531_2025_1000_MOESM1_ESM.pdf]

The mental health questionnaire includes the following questions: (1) Does your mood often go up and down? (2) Do you ever feel 'just miserable' for no reason? (3) Are you an irritable person? (4) Are your feelings easily hurt? (5) Do you often feel 'fed-up'? (6) Would you call yourself a nervous person? (7) Are you a worrier? (8) Would you call yourself tense or 'highly strung'? (9) Do you worry too long after an embarrassing experience? (10) Do you suffer from 'nerves'? (11) Do you often feel lonely? (12) Are you often troubled by feelings of guilt? (13) Would you describe yourself as someone who takes risks?

Supplementary Text S1 The information on the mental health score questionnaire.

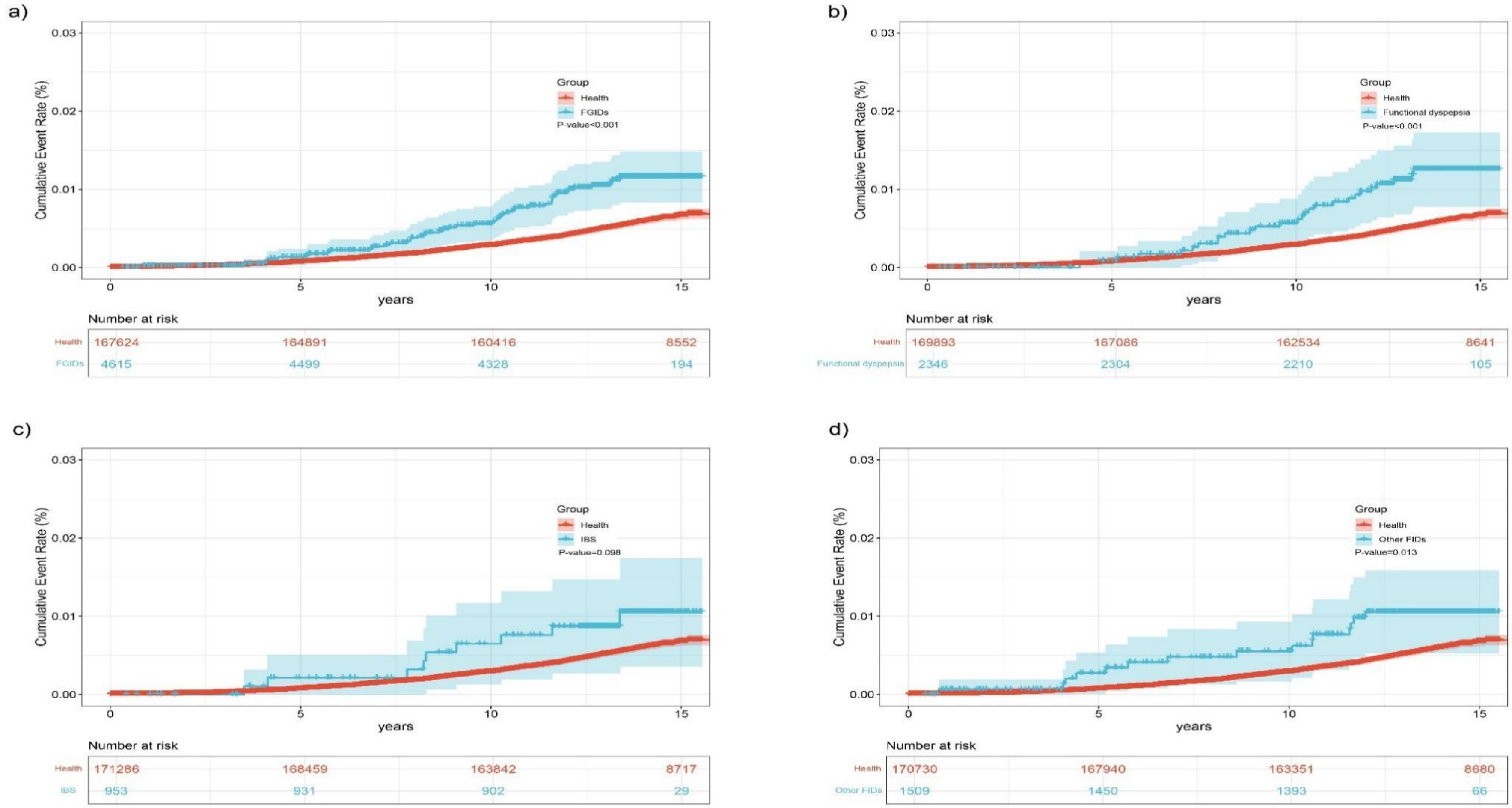

Supplementary Fig. S1 Cumulative incidence curves for Parkinson's disease in a) functional gastrointestinal disorders, b) functional dyspepsia, c) irritable bowel syndrome and d) other functional intestinal disorders and healthy population.

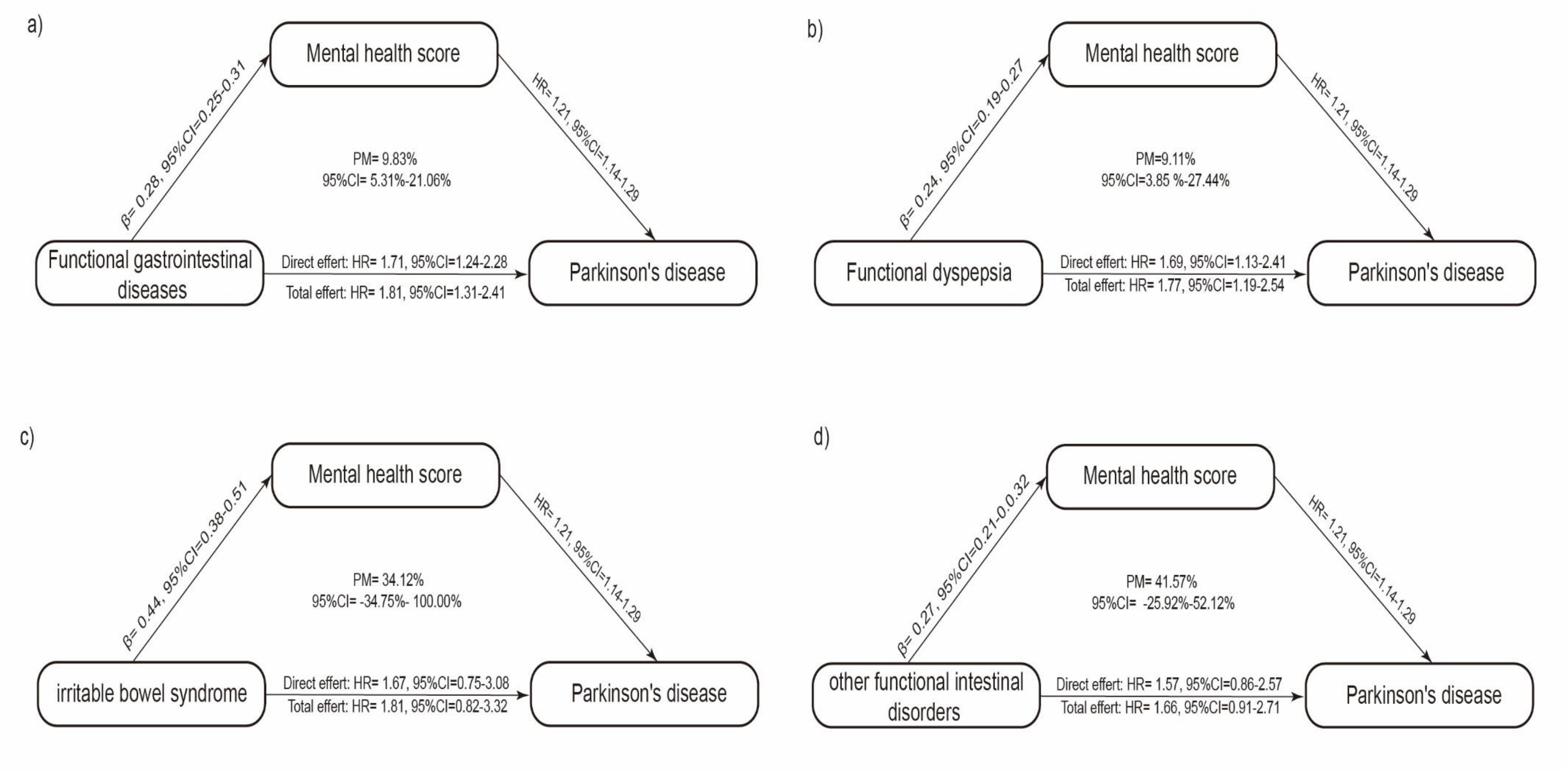

Supplementary Fig. S2 Mediation analysis of the role of mental health score in the association between a) functional gastrointestinal disorders, b) functional dyspepsia, c) irritable bowel syndrome and d) other functional intestinal disorders and the onset of Parkinson's disease.

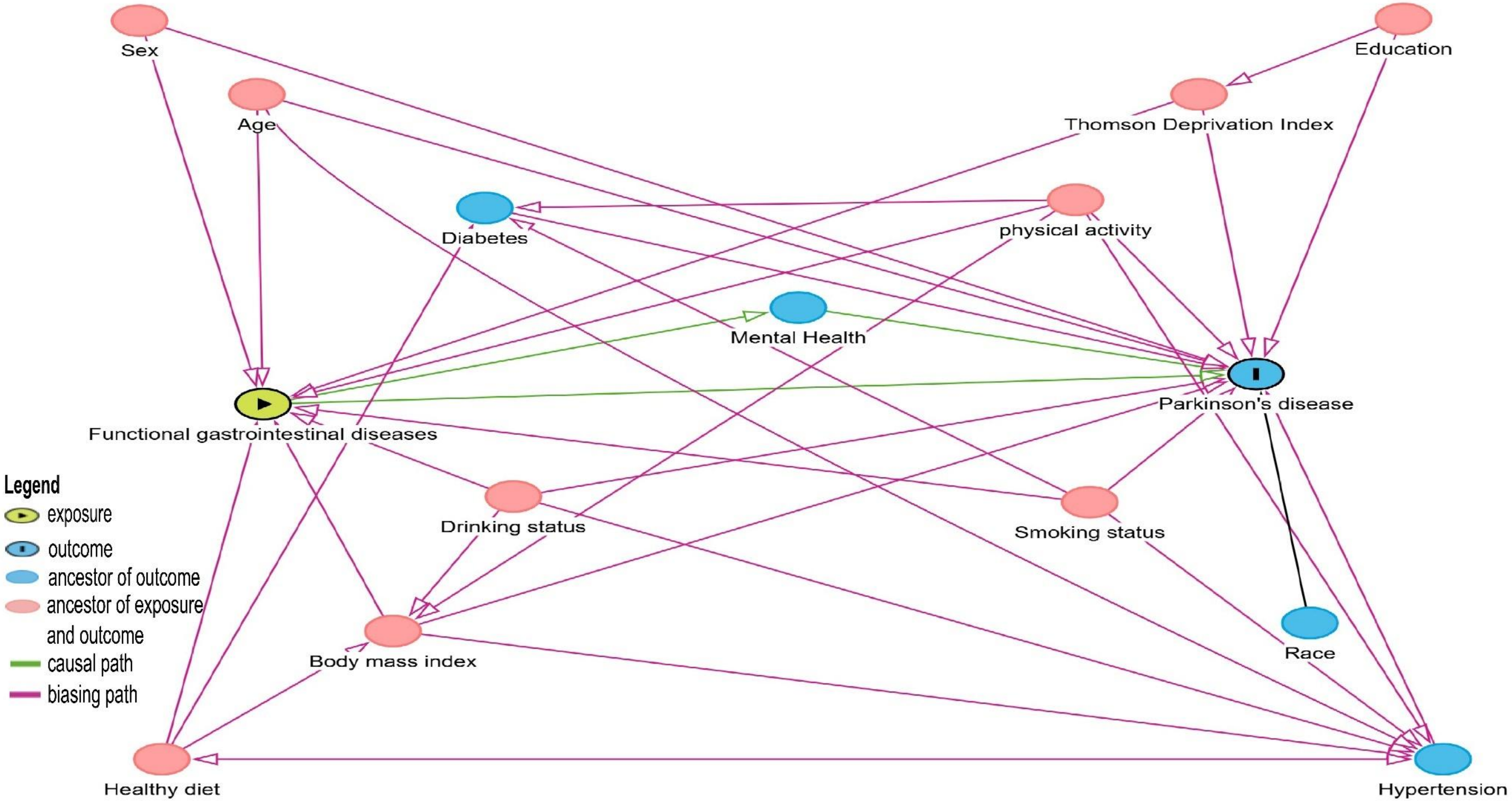

Supplementary Fig. S3 Directed acyclic graph illustrating the hypothesized causal relationship between functional gastrointestinal disorders and Parkinson's disease.

Variables	HR (95%CI)[1]	P-value	P for interaction
Sex			0.790
Male	1.78 (1.20-2.64)	0.004	
Female	1.68 (1.10-2.58)	0.017	
Age			0.197
<65	2.14 (1.49-3.09)	<0.001	
>65	1.51 (0.94-2.44)	0.089	
TDI			0.858
Low	1.82 (1.19-2.77)	0.005	
High	1.68 (1.13-2.51)	0.011	
Healthy diet score			0.737
1	0.93 (0.29-2.98)	0.903	
2	1.64 (0.84-3.21)	0.148	
3	1.69 (0.96-2.97)	0.067	
4	1.64 (0.91-2.95)	0.097	
5	2.22 (1.16-4.24)	0.016	
Drinking status			0.895
Never	1.76 (0.63-4.97)	0.282	
Previous	2.10 (0.88-4.99)	0.093	
Current	1.70 (1.23-2.34)	0.001	
Smoking status			0.810
Never	1.97 (1.26-3.07)	0.003	
Previous	1.63 (1.09-2.44)	0.018	
Current	1.45 (0.45-4.68)	0.534	
Hypertension			0.979
Normal	1.96 (1.11-3.46)	0.021	
Elevated	1.60 (0.87-2.94)	0.133	
Stage 1 hypertension	1.68 (0.82-3.44)	0.155	
Stage 2 hypertension	1.74 (1.06-2.84)	0.027	
BMI			0.598
<26	1.64 (1.02-2.64)	0.041	
>26	1.82 (1.27-2.63)	0.001	
Diabetes			0.785
No	1.77 (1.31-2.40)	<0.001	
Yes	1.39 (0.50-3.84)	0.529	
Physical activity			0.478
No	1.46 (0.74-2.87)	0.276	
Yes	1.82 (1.32-2.50)	<0.001	
Mental health score			0.269
Low	1.41 (0.85-2.32,)	0.181	
High	1.97 (1.38-2.81)	<0.001	

Supplementary Table S1. Subgroup analysis of the association between functional gastrointestinal disorders and Parkinson's disease.

[1] The HR and 95%CI represents the risk of Parkinson's disease in patients with functional gastrointestinal disorders compared to the general population within each subgroup.

Items	FGIDs		Subtypes					
			Functional dyspepsia		IBS		Other FIDs	
	No	Yes	No	Yes	No	Yes	No	Yes
Participants	167604	4614	169872	2346	171265	953	170710	1508
Number of cases	885(0.53%)	48(1.04%)	906(0.53%)	27(1.15%)	924（0.54%）	9(0.94%)	919（0.54%）	14(0.93%)
Model1	Ref	1.90 (1.42-2.55, p<.001)	Ref	1.89 (1.29-2.77, p=.001)	Ref	2.08 (1.08-4.02, p=.029)	Ref	1.74 (1.03-2.95, p=.040)
Model2	Ref	1.82 (1.36-2.43, p<.001)	Ref	1.83 (1.25-2.69, p=.002)	Ref	1.94 (1.00-3.75, p=.048)	Ref	1.65 (0.97-2.79, p=.065)
Model3	Ref	1.75 (1.30-2.34, p<.001)	Ref	1.75 (1.19-2.58, p=.004)	Ref	1.83 (0.95-3.54, p=.071)	Ref	1.59 (0.94-2.71, p=.084)

Supplementary Table S2. Associations between functional gastrointestinal disorders and Parkinson’s disease After excluding patients with Parkinson's disease of less than 2 years duration.

Items	FGIDs		Subtypes					
			Functional dyspepsia		IBS		Other FIDs	
	No	Yes	No	Yes	No	Yes	No	Yes
Participants	167553	4613	169820	2346	171214	952	170658	1508
Number of cases	834(0.50%)	47(1.02%)	854(0.50%)	27(1.15%)	873（0.51%）	8(0.84%)	867(0.51%)	14(0.93%)
Model1	Ref	1.98(1.48-2.66, p<.001)	Ref	2.01(1.37-2.95, p<.001)	Ref	1.97(0.98-3.95, p=.057)	Ref	1.85(1.09-3.15, p=.022)
Model2	Ref	1.90(1.42-2.56, p<.001)	Ref	1.95(1.33-2.86, p=.001)	Ref	1.84(0.92-3.70, p=.086)	Ref	1.76(1.04-2.99, p=.036)
Model3	Ref	1.84 (1.37-2.47, p<.001)	Ref	1.88(1.28-2.76, p=.001)	Ref	1.75(0.87-3.51, p=.116)	Ref	1.71(1.01-2.90, p=.047)

Supplementary Table S3. Associations between functional gastrointestinal disorders and Parkinson’s disease After excluding patients with Parkinson's disease of less than 4 years duration.

Items	FGIDs		Subtypes					
			Functional dyspepsia		IBS		Other FIDs	
	No	Yes	No	Yes	No	Yes	No	Yes
Participants	200536	5882	203461	2957	205195	1223	204453	1965
Number of cases	1106(0.55%)	64(1.09%)	1137(0.56%)	34(1.15%)	1158(0.56%)	13(1.06%)	1150(0.56%)	21(1.07%)
Model1	Ref	1.90(1.47-2.45, p<.001)	Ref	1.84(1.31-2.59, p=.001)	Ref	2.15(1.24-3.72, p=.006)	Ref	1.88(1.22-2.90, p=.004)
Model2	Ref	1.82(1.41-2.35, p<.001)	Ref	1.78(1.27-2.51, p=,001)	Ref	2.03(1.17-3.52, p=.011)	Ref	1.79(1.16-2.76, p=.009)
Model3	Ref	1.76(1.36-2.26, p<.001)	Ref	1.72(1.22-2.43, p=,002)	Ref	1.93(1.11-3.34, p=.019)	Ref	1.72(1.12-2.66, p=.014)

Supplementary Table S4. Associations between functional gastrointestinal disorders and Parkinson's disease when adding participants with missing data on physical activity

Items	FGIDs		Subtypes					
			Functional dyspepsia		IBS		Other FIDs	
	No	Yes	No	Yes	No	Yes	No	Yes
Participants	154008	4615	166643	2346	167923	953	162025	1509
Number of cases	621(0.40%)	49(1.06%)	891(0.53%)	27(1.15%)	914(0.54%)	9(0.94%)	674(0.42%)	15(0.99%)
Model1	Ref	2.56 (1.91-3.43, p<.001)	Ref	1.88 (1.28-2.76, p=.001)	Ref	2.07 (1.07-3.99, p=.031)	Ref	2.43 (1.46-4.06, p=.001)
Model2	Ref	2.44 (1.82-3.28, p<.001)	Ref	1.83 (1.25-2.69, p=.002)	Ref	1.93 (1.00-3.72, p=.051)	Ref	2.29 (1.37-3.83, p=.002)
Model3	Ref	2.31 (1.72-3.11, p<.001)	Ref	1.75 (1.19-2.57, p=.004)	Ref	1.78 (0.92-3.44, p=.087)	Ref	2.17 (1.30-3.63, p=.003)

Supplementary Table S5. Association between functional gastrointestinal disorders and Parkinson's disease after removal of participants with functional gastrointestinal disorders after baseline.

PRS Subgroups	Diseases	HR (95%CI)	P-value	P for interaction (When PRS is the categorical variable)[1]	P for interaction (When PRS is the continuous variable)[2]
	FGIDs			0.636	0.668
Low	No	Ref			
Low	Yes	1.56 (0.94-2.58)	0.087		
High	No	Ref			
High	Yes	1.91 (1.28-2.84)	0.001		
	Functional dyspepsia			0.704	0.340
Low	No	Ref			
Low	Yes	1.59 (0.82-3.09)	0.171		
High	No	Ref			
High	Yes	1.97 (1.18-3.30)	0.010		
	IBS			0.294	0.446
Low	No	Ref			
Low	Yes	1.10 (0.27-4.42)	0.895		
High	No	Ref			
High	Yes	2.49 (1.11-5.59)	0.027		
	Other FIDs			0.260	0.547
Low	No	Ref			
Low	Yes	2.11 (0.99-4.47)	0.052		
High	No	Ref			
High	Yes	1.16 (0.48-2.80)	0.747		

Supplementary Table S6. Association between a) functional gastrointestinal disorders, b) functional dyspepsia, c) irritable bowel syndrome and d) other functional intestinal disorders and Parkinson's disease after dividing participants into two groups using polygenic risk score for Parkinson's disease.

[1] The P for the interaction was calculated as a dichotomous variable form of PRS interacting with FGIDs and their subtypes

[2] The P for the interaction was calculated as a continuous variable form of PRS interacting with FGIDs and their subtypes

Items	FGIDs		Subtypes					
			Functional dyspepsia		IBS		Other FIDs	
	No	Yes	No	Yes	No	Yes	No	Yes
Participants (Stage 1)[1]	167624	4615	169893	2346	171286	953	170730	1509
Participants (Stage 2)[1]	161659	10580	167464	4775	170080	2159	167830	4409
Number of cases	905（0.54%）	49（1.06%）	927（0.55%）	27（1.15%）	945（0.55%）	9（0.94%）	939（0.55%）	15（0.99%）
Model1	Ref	1.92 (1.55-2.38, p<.001)	Ref	1.98 (1.49-2.63, p<.001)	Ref	1.93 (1.19-3.12, p=.008)	Ref	1.88 (1.35-2.60, p<.001)
Model2	Ref	1.85 (1.49-2.29, p<.001)	Ref	1.93 (1.45-2.56, p<.001)	Ref	1.81 (1.12-2.94, p=.016)	Ref	1.79 (1.29-2.49, p<.001)
Model3	Ref	1.78 (1.44-2.20, p<.001)	Ref	1.86 (1.40-2.47, p<.001)	Ref	1.71 (1.06-2.77, p=.030)	Ref	1.73 (1.24-2.40, p=.001)

Supplementary Table S7. Time-dependent association between functional gastrointestinal disorders and Parkinson's disease by the time-dependent Cox proportional hazards models.

[1]Stage 1 was from the time of enrolment to 1 January 2016, and Stage 2 was from 1 January 2016 to the end of the follow-up.

Covariate	Start date of baseline assessment	End data of baseline assessment
Age	April 2007	July 2010
Sex		
Race		
Smoking status		
Drinking status		
Educational level		
Physical activity		
Townsend Deprivation Index		
Diabetes		
Hypertension		
Body mass index		
Mental health score		
Healthy diet score	April 2009	
Polygenic risk score	August 2009	

Supplementary Table S8. The start and end time of baseline assessment for each covariate

Diseases	Time-by-Exposure Interaction Test[1]	Schoenfeld Residual test
	P-value	P-value
FGIDs	0.496	0.286
Functional dyspepsia	0.894	0.783
IBS	0.468	0.373
Other FIDs	0.109	0.122

Supplementary Table S9. Proportional hazards (PH) assumption assessment for univariate Cox regression models evaluating associations between functional gastrointestinal disorders and their subtypes with PD.

[1] The time-exposure interaction test assesses the proportional hazards hypothesis by evaluating the significance of the interaction term between time t and the exposure variable.

	Item No	Recommendation	Page/line No[#]
**Title and abstract**	1	(*a*) Indicate the study's design with a commonly used term in the title or the abstract	2/33-44
		(*b*) Provide in the abstract an informative and balanced summary of what was done and what was found	2/35-44
**Introduction**			
Background/rationale	2	Explain the scientific background and rationale for the investigation being reported	3-5/49-91
Objectives	3	State specific objectives, including any prespecified hypotheses	5/92-98
**Methods**			
Study design	4	Present key elements of study design early in the paper	12/253-261
Setting	5	Describe the setting, locations, and relevant dates, including periods of recruitment, exposure, follow-up, and data collection	12/253-261
Participants	6	(*a*) Give the eligibility criteria, and the sources and methods of selection of participants. Describe methods of follow-up	12-13/262-265
		(*b*) For matched studies, give matching criteria and number of exposed and unexposed	NA
Variables	7	Clearly define all outcomes, exposures, predictors, potential confounders, and effect modifiers. Give diagnostic criteria, if applicable	13-15/266-324
Data sources/ measurement	8*	For each variable of interest, give sources of data and details of methods of assessment (measurement). Describe comparability of assessment methods if there is more than one group	13-15/266-324
Bias	9	Describe any efforts to address potential sources of bias	17-18/363-386

Study size	10	Explain how the study size was arrived at	12-13/262-265
Quantitative variables	11	Explain how quantitative variables were handled in the analyses. If applicable, describe which groupings were chosen and why	14-15/298-324
Statistical methods	12	(*a*) Describe all statistical methods, including those used to control for confounding	15-17/326-362
		(*b*) Describe any methods used to examine subgroups and interactions	17/367-368
		(*c*) Explain how missing data were addressed	17/369-373
		(*d*) If applicable, explain how loss to follow-up was addressed	NA
		(*e*) Describe any sensitivity analyses	17-18/363-386
**Results**			
Participants	13*	(a) Report numbers of individuals at each stage of study—eg numbers potentially eligible, examined for eligibility, confirmed eligible, included in the study, completing follow-up, and analysed	12-13/262-265
		(b) Give reasons for non-participation at each stage	12-13/262-265
		(c) Consider use of a flow diagram	12-13/262-265 (Fig,3)
Descriptive data	14*	(a) Give characteristics of study participants (eg demographic, clinical, social) and information on exposures and potential confounders	5/101-107
		(b) Indicate number of participants with missing data for each variable of interest	12-13/262-265
		(c) Summarise follow-up time (eg, average and total amount)	5/101-107
Outcome data	15*	Report numbers of outcome events or summary measures over time	5/101-107
Main results	16	(*a*) Give unadjusted estimates and, if applicable, confounder-adjusted estimates and their precision (eg, 95% confidence interval). Make clear which confounders were adjusted for and why they were included	5-7/108-136
		(*b*) Report category boundaries when continuous variables were categorized	7/142-151
		(*c*) If relevant, consider translating estimates of relative risk into absolute risk for a meaningful time period	NA

Other analyses	17	Report other analyses done—eg analyses of subgroups and interactions, and sensitivity analyses	7-8/137-168
**Discussion**			
Key results	18	Summarise key results with reference to study objectives	8/170-176
Limitations	19	Discuss limitations of the study, taking into account sources of potential bias or imprecision. Discuss both direction and magnitude of any potential bias	11-12/228-243
Interpretation	20	Give a cautious overall interpretation of results considering objectives, limitations, multiplicity of analyses, results from similar studies, and other relevant evidence	12/244-249
Generalisability	21	Discuss the generalisability (external validity) of the study results	10/204-211 and 11/228-230
**Other information**			
Funding	22	Give the source of funding and the role of the funders for the present study and, if applicable, for the original study on which the present article is based	19/398-402

Supplementary Table S10. STROBE Statement Checklist

*Give information separately for exposed and unexposed groups.

[#]The number before / represents the number of pages, the number after / represents the number of lines.
